# Nonadiabaticity in Quantum Pumping Phenomena under Relaxation

**DOI:** 10.3390/e21090842

**Published:** 2019-08-28

**Authors:** Kazunari Hashimoto, Chikako Uchiyama

**Affiliations:** 1 Faculty of Engineering, University of Yamanashi, 4-3-11 Takeda, Kofu 400-8511, Japan; 2 National Institute of Informatics, 2-1-2 Hitotsubashi, Chiyoda-ku, Tokyo 101-8430, Japan

**Keywords:** nonadiabaticity, quantum heat pumping, spin pumping, relaxation

## Abstract

The ability to control quanta shown by quantum pumping has been intensively studied, aiming to further develop nano fabrication. In accordance with the fast progress of the experimental techniques, the focus on quantum pumping extends to include the quicker transport. For this purpose, it is necessary to remove the “adiabatic” or “slow” condition, which has been the central concept of quantum pumping since its first proposal for a closed system. In this article, we review the studies which go beyond the conventional adiabatic approximation for open quantum systems to transfer energy quanta and electron spins with using the full counting statistics. We also discuss the recent developments of the nonadiabatic treatments of quantum pumping.

## 1. Introduction

According to the rapid development of experimental techniques, the downsizing of devices has been accelerated to extend possibilities to control single-electron current [1], spin-polarized current [2,3] and even thermal transport [4]. The trend is based on the aim to construct electronics with low energy consumption, quantum information processing, as well as quantum metrology [1,5].

Quantum pumping phenomena have attracted intensive attention, since they show the controllability of quantum transfer to extend the possibility of nano fabrications. The first proposal of quantum pumping was given by Thouless to transport electrons between two environments [6,7]. Its essential point is to adiabatically or slowly modulate the potential, which is described with the superposition of two standing waves in an out-of phase way [6,7,8]. Since the work of Thouless, the “adiabatic” change or “slow” modulation of parameters has played a central role in theoretical treatments of quantum pumping phenomena. However, the fast development of experimental techniques after the first experimental study on electron pumping with a quantum dot [9] requires us to investigate conditions on transferring quanta quicker and more precisely. In the present review, we classify the meaning of “quick” or “slow” in quantum pumping and show a standardized theoretical treatment—called full counting statistics (FCS)—to attain the purpose.

The physical situations referred to by the same term “adiabaticity” are roughly divided into three categories: (1) slow change in potential to allow the application of the adiabatic approximations to wave functions associated with transported particles [6,7]; (2) slow and small change of parameter(s) such as the chemical potential and voltage to allow a linear expansion of the scattering matrix [10,11,12,13,14,15,16,17] or Green functions [18,19,20,21,22,23,24,25,26] associated with transported electron charge or spin; and (3) slow change of parameters compared with the relaxation time of the relevant system with using FCS [27,28,29,30,31,32]. Different from the former two treatments, the third succeeds in including explicitly the finite relaxation time in adiabaticity. Because isolating any quantum system from its surroundings is impossible, considering relaxation phenomena is indispensable in implementing quantum pumping systems. With further developments of experimental techniques in mind, removing the adiabatic condition in this third instance is necessary. Before going further, let us provide a quick review of the conventional studies on pumping phenomena from the view point of the above summarized classifications of the adiabaticity.

Sinitsyn and Nemenman treated the classical two-state stochastic system [27] using FCS to represent the pumped quantity with a geometrical phase, first expounded in Reference [33]. The relationship between finite relaxation time and adiabaticity in the context of open quantum systems was first discussed by Ren, Hänggi, and Li [28] by extending the FCS approach in Reference [27]. Considering a two-level system coupled to two environments, they found that the pumping of energy quanta occurs under out-of-phase conditions and sufficiently slow (adiabatic) temperature modulations of the environments even if the bias is averaged out during a period. The power of the FCS approach can be found in the further application to electron charge pumping through single or double quantum dot(s) coupled to two leads [29,30,31]. They found that the sufficiently slow and out-of-phase modulations of the chemical potential can induce electron charge pumping, which is represented by a geometrical formula. In these instances, the condition for a sufficiently slow (adiabatic) environmental modulation means that the relevant two-level system approaches steady state sufficiently quickly.

In many varieties of quantum pumping phenomena, the generation of spin polarized electron current (spin current) by periodic parameter modulation has attracted a great deal of attention because of its promising applications in spintronics. Referred to as spin pumping, much effort has been made to develop its protocols. Conventionally, the protocols fall roughly into three classes: those using (i) a precession of magnetization in a magnetic material attached to a normal metal [12,13,19,20,21,22,23,24,25,26,34,35,36]; (ii) a periodic modulation of parameters such as gate voltages and/or tunneling amplitudes in a system consisting of quantum dots subjected to a magnetic field and normal metal leads [14,32,37,38,39,40]; and (iii) a periodic modulation of strength of magnetization in addition to parameters in a system consisting of quantum dots attached to a magnetic lead and/or normal metal leads [34,35,41,42]. Among these protocols, those using precession of magnetization—protocol (i)—have attracted intensive studies because it can generate pure spin current in a simple ferromagnet/normal metal heterojunction [3]. So far, the protocol has been mostly studied in situations where the precession of the magnetization is sufficiently slow, which is called adiabatic pumping. It was first proposed by Tserkovnyak et al. [12,13] based on the scattering theory of adiabatic quantum pumping given by Brouwer [11]. Its alternative formalisms based on Green’s function [19,20,22,23,24,25,26,36] have also been proposed by several authors. In these studies, the adiabatic contribution to the spin current generation has been obtained as a linear response to the precession, which corresponds to adiabaticity No. (2), with an implicit assumption of an infinite relaxation time. There are a few studies addressing adiabatic spin pumping with a finite relaxation time [34,35], where a slow modulation means smallness of the precession frequency comparing with the tunneling rate.

In the present article, we intend to review our recent studies on the role of nonadiabaticity with a finite relaxation time in quantum pumping of energy quanta and electron spins. For the purpose, we rely on adiabaticity condition No. (3), where the adiabaticity means a slow modulation of parameters compared to the relaxation time of the relevant system—defining the relaxation time of the relevant system as $\tau_{r}$, we find that the condition for a slow modulation requires $\tau_{r}$ to be much shorter than the period of the temperature modulation, which is written equivalently in terms of the modulation frequency Ω, $\tau_{r}^{-1}\gg \Omega$. Thus, we consider the nonadiabatic regime up to $\tau_{r}^{-1}≳\Omega$ in the following. As a formulation of nonadiabatic pumping, we present our extension of the FCS approach to quantum pumping toward the nonadiabatic regime. By applying the formulation to the pumping phenomena of energy quanta and electron spin, we find the following features: For the former, we demonstrate that nonadiabaticity yields a contribution to the pumped quantity in addition to the terms such as dynamical and geometrical phase terms which were obtained under adiabatic conditions. For the latter, surprisingly, we show that there are no contributions under the adiabatic condition and nonadiabaticity is an essential feature.

In the rest of the paper, we present in Section 2 our formulation describing the pumped quantity based on FCS. We discuss quantum heat pumping in Section 3 and spin pumping in Section 4, followed by a discussion and conclusion in Section 5.

## 2. General Formalism

We formulate a model of quantum pumping under periodic modulation of a parameter applying the FCS based on two-point projective measurements [43,44]. Let us consider a system consisting of a relevant system (S) and an environment (E) described by the Hamiltonian

(1) $$H=H_{0}+H_{\mathrm{SE}},$$

where $H_{0}\equiv H_{S}+H_{E}$ and $H_{\mathrm{SE}}$ is the system–environment interaction. The FCS provides the statistical average of the net amount of a physical quantity, such as energy and particle number, exchanged between the system and the environment during a certain time interval. It is based on a joint probability of outcomes of two successive projective measurements of an observable of the environment *Q* corresponding to the exchanged quantity. The measurement scheme is—at $t=t_{i}$, we perform a projective measurement of *Q* to obtain a measurement outcome $q_{t_{i}}$; for $t_{i}\leq t\leq t_{i+1}$, the system undergoes a unitary time evolution through an interaction between the system and the environment; and at $t=t_{i+1}$, we perform a second projective measurement of *Q* to obtain another outcome $q_{t_{i+1}}$. The joint probability for the measurement scheme is given by

(2) $$P\left[q_{t_{i+1}},q_{t_{i}}\right]\equiv \mathrm{Tr}\left[P_{q_{t_{i+1}}}U\left(t_{i+1},t_{i}\right)P_{q_{t_{i}}}W\left(t_{i}\right)P_{q_{t_{i}}}U^{†}\left(t_{i+1},t_{i}\right)P_{q_{t_{i+1}}}\right],$$

where Tr denotes the trace taken over the total system, $P_{q_{t}}\equiv |q_{t}\rangle \;\langle q_{t}|$ the projective measurement of *Q* at *t*, $U(t,t_{i})$ the unitary time evolution operator for the total system, and $W(t_{i})$ the initial condition of the total system (see Note [45]). The net amount of exchanged quantity during the time interval $\delta t\equiv t_{i+1}-t_{i}$ is then given by $\Delta q_{i}\equiv q_{t_{i+1}}-q_{t_{i}}$, where its sign is chosen to be positive when the physical quantity is transferred from the system to the environment. The statistics of $\Delta q_{i}$ is contained in its probability distribution function

(3) $$P\left(\Delta q_{i}\right)\equiv \sum_{q_{t_{i+1}},q_{t_{i}}}\delta \left(\Delta q_{i}-\left(q_{t_{i+1}}-q_{t_{i}}\right)\right)P\left[q_{t_{i+1}},q_{t_{i}}\right],$$

The moments of $\Delta q_{i}$ are provided by the moment generating function,

(4) $$Z\left(\lambda \right)\equiv \int_{-\infty }^{\infty }P\left(\Delta q_{i}\right)e^{i\lambda \Delta q_{i}}d\Delta q_{i}.$$

where λ is the counting field associated with *Q*, for example, the mean value is computed from

(5) $$\langle \Delta q_{i}\rangle =\frac{\partial Z(\lambda )}{\partial (i\lambda )}{|}_{\lambda =0}.$$

Our next task is to describe the time evolution of Z(λ). Using the Definition (3) and introducing the modified evolution operator $U_{\lambda }\left(t,t_{i}\right)\equiv e^{i\lambda Q}U\left(t,t_{i}\right)e^{-i\lambda Q}$, the moment generating function Z(λ) is expressed as

(6) $$Z\left(\lambda \right)=\mathrm{Tr}[W^{\left(\lambda \right)}\left(t_{i+1}\right)],$$

with

(7) $$W^{\left(\lambda \right)}\left(t\right)\equiv U_{\lambda /2}\left(t,t_{i}\right)\bar{W}\left(t_{i}\right)U_{-\lambda /2}^{†}\left(t,t_{i}\right),$$

where $\bar{W}\left(t_{i}\right)\equiv \sum_{q_{t_{i}}}P_{q_{t_{i}}}W\left(t_{i}\right)P_{q_{t_{i}}}$ is the diagonal part of $W(t_{i})$. For λ=0, $W^{\left(\lambda =0\right)}\left(t\right)$ reduces to the usual reduced density matrix of the total system. By taking the time derivative of $W^{\left(\lambda \right)}\left(t\right)$, we obtain a modified Liouville–von Neumann equation

(8) $$i\frac{\partial }{\partial t}W^{\left(\lambda \right)}\left(t\right)=L^{\left(\lambda \right)}W^{\left(\lambda \right)}\left(t\right),$$

with a modified Liouvillian $L^{\left(\lambda \right)}W^{\left(\lambda \right)}\left(t\right)\equiv \hbar^{-1}{\left[H,W^{\left(\lambda \right)}\left(t\right)\right]}_{\lambda }$, where ${\left[A,B\right]}_{\lambda }\equiv A^{\left(\lambda \right)}B-BA^{\left(-\lambda \right)}$, and $A^{\left(\lambda \right)}\equiv e^{i\lambda Q/2}Ae^{-i\lambda Q/2}$. In Appendix B, we explain the connection between the formalism of the FCS based on two-point measurements and the formalism by Sinitsyn and Nemenman in Reference [27].

By introducing a projection operator $P:W^{\left(\lambda \right)}\left(t\right)\mapsto {\mathrm{Tr}}_{E}\left[W^{\left(\lambda \right)}\left(t\right)\right]⊗\rho_{E}$, where ${\mathrm{Tr}}_{E}$ is the partial trace taken over the environment and $\rho_{E}$ is a fixed state of the environment, the equation of motion for the reduced operator of the relevant system $\rho^{\left(\lambda \right)}\left(t\right)\equiv {\mathrm{Tr}}_{E}\left[W^{\left(\lambda \right)}\left(t\right)\right]$ can be cast into the form of a time convolutionless (TCL)-type quantum master equation [46,47,48,49,50,51,52,53].

Assuming that the initial state is factorized between system and environment as $W\left(t_{i}\right)=\rho \left(t_{i}\right)⊗\rho_{E}$ and the fixed state of the environment $\rho_{E}$ is the Gibbs state with an inverse temperature β, the TCL master equation including the counting field is expressed as

(9) $$\frac{\partial }{\partial t}\rho^{\left(\lambda \right)}\left(t\right)=\xi^{\left(\lambda \right)}\left(t\right)\rho^{\left(\lambda \right)}\left(t\right).$$

The super-operator $\xi^{\left(\lambda \right)}\left(t\right)$ is expanded as a sum of “ordered cumulants” of the interaction Hamiltonian $H_{\mathrm{SE}}$ up to infinite order. Taking leading terms up to second-order, we have

(10) $$\xi^{\left(\lambda \right)}\left(t\right)\rho^{\left(\lambda \right)}\left(t\right)=-\frac{i}{\hbar }\left[H_{S},\rho^{\left(\lambda \right)}\left(t\right)\right]-\frac{1}{\hbar^{2}}\int_{0}^{t}d\tau {\mathrm{Tr}}_{E}{\left[H_{\mathrm{SE}},{\left[H_{\mathrm{SE}}\left(-\tau \right),\rho^{\left(\lambda \right)}\left(t\right)⊗\rho_{E}^{\mathrm{eq}}\right]}_{\lambda }\right]}_{\lambda }.$$

Note that the time dependence of the memory kernel reflects the finiteness of the correlation time of the dot–lead interaction, which allows us to describe the non-Markovian dynamics.

To work with the super-operator, it is convenient to introduce its supermatrix representation, where we represent the density matrix $\rho^{\left(\lambda \right)}$ in vector form and the super-operator $\xi^{\left(\lambda \right)}$ in matrix form. In this representation, the formal solution of the master equation Equation (9) is expressed as

(11) $$|\rho^{\left(\lambda \right)}\left(t\right)\rangle \;\rangle =T_{+}\exp[\int_{t_{i}}^{t}\Xi^{\left(\lambda \right)}\left(s\right)ds]|\rho^{\left(\lambda \right)}\left(t_{i}\right)\rangle \rangle ,$$

where $|\rho^{\left(\lambda \right)}\left(t\right)\rangle \;\rangle$ represents the vector form of $\rho^{\left(\lambda \right)}\left(t\right)$, $T_{+}\exp$ the time-ordered exponential, and $\Xi^{\left(\lambda \right)}\left(t\right)$ the supermatrix form of $\xi^{\left(\lambda \right)}\left(s\right)$. With the representation, the moment generating function Equation (6) is rewritten as $Z\left(\lambda \right)={\mathrm{Tr}}_{S}\left[\rho^{\left(\lambda \right)}\left(t_{i+1}\right)\right]=\langle \;\langle 1|\rho^{\left(\lambda \right)}\left(t_{i+1}\right)\rangle \;\rangle$, where ${\mathrm{Tr}}_{S}$ is the partial trace taken over the relevant system and 〈〈1| the vector representation of the partial trace ${\mathrm{Tr}}_{S}$. Using the formal solution Equation (11), we recast the expression of the mean value into the form

(12) $$\langle \Delta q_{i}\rangle =\int_{t_{i}}^{t_{i+1}}J\left(s\right)ds$$

with the inertial flow of the quantity,

(13) $$J\left(t\right)\equiv \langle \;\langle 1|[\frac{\partial \Xi^{\left(\lambda \right)}\left(t\right)}{\partial (i\lambda )}{]}_{\lambda =0}|\rho^{\left(\lambda =0\right)}\left(t\right)\rangle \;\rangle .$$

To formulate quantum pumping based on the above framework, we need to accumulate transfers of the physical quantity under a cyclic modulation of system and/or environmental parameters during a period T. For this purpose, we consider a step-like change of the parameters; specifically, dividing the period T into *N* intervals, $t_{i}\leq t\leq t_{i+1}$ (i=1,2,⋯,N) with $t_{1}=0$ and $t_{N+1}=T$, fixing a value of the parameters during each interval, and changing the value at each $t_{i}$ discretely. With the total density matrix factorized at each $t_{i}$, the mean value as well as the inertial flow of the quantity for each interval are given by Equations (12) and (13), respectively. The time integration of J(t) over one period T provides the accumulated value of the quantity over one cycle

(14) $$\langle \Delta q\rangle \equiv \int_{0}^{T}J\left(t\right)dt=\sum_{i=1}^{N}\langle \Delta q_{i}\rangle ,$$

where $\langle \Delta q_{i}\rangle$ is the mean value of the net transferred quantity in the *i*th interval.

## 3. Pumping of Energy Quanta

### 3.1. Model

**Figure 1:**
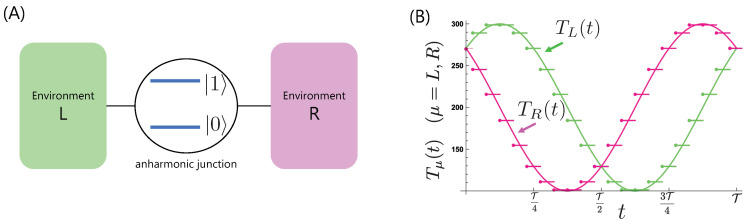
(**A**) Model scheme: a two-level system as an anharmonic junction interacts with two environments (*L* and *R*). (**B**) Temperature modulations, $T_{L}\left(t\right)=200+100\cos\left(\omega t+\pi /4\right)$, and $T_{R}\left(t\right)=200+100\sin\left(\omega t+\pi /4\right)$, discretized with N=20.

Let us consider the energy transfer via a two-level system between two environmental systems (*L* and *R*) consisting of an infinite number of bosons [28,54,55,56]. With the definition of the lower (higher) level of the two-level system as |0〉 (|1〉), respectively, the Hamiltonian of the model is written as

(15) $$\begin{array}{ccc} H_{S} & = & \sum_{m=0,1}\varepsilon_{m}|m\rangle \langle m|,\;\;H_{E}=\sum_{k,\nu }\hbar \omega_{k,\nu }b_{k,\nu }^{†}b_{k,\nu },\\ H_{SE} & = & \sum_{\nu }X_{\nu }(|0\rangle \langle 1|+|1\rangle \langle 0|),\;\left(\nu =L,R\right), \end{array}$$

where $X_{\nu }=\sum_{k}\hbar g_{k,\nu }\left(b_{k,\nu }^{†}+b_{k,\nu }\right)$, with $b_{k,\nu }^{†}$ and $b_{k,\nu }$ the creation and annihilation boson operators of the *k*th mode of the νth environment. The model scheme is shown in Figure 1A.

We consider the energy transfer from out-of-phase temperature modulations of the two environments, corresponding to the bias averaged out during a period T [28]. To discuss the nonadiabaticity for this model, we study energy (boson) transfers under cyclic and piecewise modulations of the environmental temperatures $T_{L}$ and $T_{R}$ dividing T into *N* intervals, $t_{i}\leq t\leq t_{i+1}$(i=1,⋯,N) with $t_{1}=0$ and $t_{N+1}=T$. We need to discretize the temperature modulation because conventional treatments describing relaxation phenomena require the environmental temperature to remain constant. By changing the number of intervals of the temperature modulation, we compared each time interval with the relaxation time of the relevant two-level system and thus we are able to discuss nonadiabaticity explicitly, for example, from the scale between $\tau_{r}^{-1}$ and Ω. In taking the limit N→∞, we reveal energy transfer features under a continuous modulation. In Figure 1B, we plot the time dependence of the temperature modulations used in this study calculated for a typical number of discrete time intervals N=20.

### 3.2. FCS Formalism Applied to Pumping of Energy Quanta

We apply the general formalism of the FCS in the former section to this model focusing on weak system–environment coupling and considering long time (Born-Markovian) limits by taking t→∞ in the upper bound of the integral in Equation (10). In this limit, the super-operator $\xi^{\left(\lambda \right)}\left(t\right)$ becomes time independent during each interval. We find that the mean value of the transferred quantity between the relevant two-level system and the νth environment in the *i*th time interval, $\langle \Delta q_{i}^{\nu }\rangle$ is written as

(16) $$\begin{array}{c} \langle \Delta q_{i}^{\nu }\rangle =\hbar \omega_{0}\left\{A_{i}^{\nu }\int_{t_{i}}^{t_{i+1}}dt^{\prime }\rho_{00}\left(t^{\prime }\right)-B_{i}^{\nu }\delta t\right\}, \end{array}$$

with $\omega_{0}=\left(\varepsilon_{1}-\varepsilon_{0}\right)/\hbar$, $\rho_{00}\left(t\right)=\langle 0|\rho^{\left(\lambda =0\right)}\left(t\right)|0\rangle$, and $\delta t=t_{i+1}-t_{i}$. $A_{i}^{\nu }$ and $B_{i}^{\nu }$ in Equation (16) are coefficients defined as $A_{i}^{\nu }=-\left(k_{d,i}^{\nu }+k_{u,i}^{\nu }\right)$ and $B_{i}^{\nu }=-k_{u,i}^{\nu }$ with rate constants $k_{u,i}^{\nu }$ and $k_{d,i}^{\nu }$, which govern the time evolution of $\rho_{00}\left(t\right)$ during $t_{i-1}\leq t<t_{i}$. Their explicit expressions are $k_{d,i}^{\nu }=\Gamma_{\nu }n_{\nu ,i}$ and $k_{u,i}^{\nu }=\Gamma_{\nu }\left(1+n_{\nu ,i}\right)$, where $n_{\nu ,i}=1/\left(\exp\left[\hbar \beta_{i}^{\nu }\omega_{0}\right]-1\right)$ is the Bose–Einstein distribution for the inverse temperature $\beta_{i}^{\nu }$ of the νth environment during the *i*th interval and $\Gamma_{\nu }$ denotes the feature of the system–environment coupling as $\Gamma_{\nu }=2\pi h_{\nu }\left(\omega_{0}\right)$ with the coupling spectral density $h_{\nu }\left(\omega \right)\equiv \sum_{k}g_{k,\nu }^{2}\delta \left(\omega -\omega_{k,\nu }\right)=\lambda \omega \exp\left[-\omega /\omega_{c}\right]$, where λ is the coupling strength and $\omega_{c}$ is the cutoff frequency. To obtain $\langle \Delta q_{i}^{\nu }\rangle$, we need $\rho_{00}\left(t\right)$, the time evolution for which is

$$\begin{array}{c} {\dot{\rho }}_{00}\left(t\right)=-K_{d,i}\rho_{00}\left(t\right)+K_{u,i}\rho_{11}\left(t\right) \end{array}$$

with $K_{d,i}=\sum_{\nu }k_{d,i}^{\nu }$ and $K_{u,i}=\sum_{\nu }k_{u,i}^{\nu }$. The differential equation for $\rho_{00}\left(t\right)$ is solved to give

(17) $$\begin{array}{c} \rho_{00}\left(t\right)=\rho_{s,i}+e^{\Lambda_{i}t}\left(\rho_{00}\left(t_{i-1}\right)-\rho_{s,i}\right), \end{array}$$

where we denote $\rho_{s,i}=-K_{u,i}/\Lambda_{i}$ with $\Lambda_{i}=-\left(K_{d,i}+K_{u,i}\right)$. Using Equations (12), we find that the total transferred energy during the period is calculated to be

(18) $$\begin{array}{c} \langle \Delta q^{\nu }\rangle =\sum_{i=1}^{N+1}\langle \Delta q_{i}^{\nu }\rangle =\hbar \omega_{0}\left(G_{1}^{\nu }+G_{2}^{\nu }+G_{3}^{\nu }\right),\;\; \end{array}$$

where

(19) $$\begin{array}{ccc} G_{1}^{\nu } & = & \sum_{i=1}^{N+1}\left(A_{i}^{\nu }\rho_{s,i}-B_{i}^{\nu }\right)\delta t, \end{array}$$

(20) $$\begin{array}{ccc} G_{2}^{\nu } & = & \sum_{i=1}^{N}\frac{A_{i+1}^{\nu }}{\Lambda_{i+1}}\left(\rho_{s,i+1}-\rho_{s,i}\right),\; \end{array}$$

(21) $$\begin{array}{ccc} G_{3}^{\nu } & = & \sum_{i=1}^{N+1}\phi_{0,i}^{\nu }+\sum_{i=2}^{N}\left(\rho_{s,i-1}-\rho_{s,i}\right)\psi_{i}^{\nu }+\left(\rho_{s,n-1}-\rho_{s,n}\right)\frac{A_{n}^{\nu }}{\Lambda_{n}}e^{\Lambda_{n}\delta t}, \end{array}$$

with

(22) $$\begin{array}{ccc} \phi_{0,i}^{\nu } & = & \left(\rho_{00}\left(0\right)-\rho_{s,1}\right)f^{\nu }\left(1,i\right), \end{array}$$

(23) $$\begin{array}{ccc} \psi_{i}^{\nu } & = & \frac{A_{i}^{\nu }}{\Lambda_{i}}e^{\Lambda_{i}\delta t}+\sum_{m=i}^{N}f^{\nu }\left(i,m+1\right), \end{array}$$

(24) $$\begin{array}{ccc} f^{\nu }\left(p,q\right) & = & \frac{A_{q}^{\nu }}{\Lambda_{q}}e^{\sum_{\kappa =p}^{q-1}\Lambda_{\kappa }\delta t}\left(e^{\Lambda_{q}\delta t}-1\right). \end{array}$$

In the next subsection, we show the physical meanings of these obtained terms.

### 3.3. Adiabatic and Nonadiabatic Contributions

Taking the Riemann sum on $G_{1}^{\nu }$ and $G_{2}^{\nu }$ by setting N→∞ and δt→0, we find that they reduce to the dynamical and geometrical phases, respectively. For instance, we obtain the energy transfer with the environment R with setting ν=R as [56]

(25) $$G_{1}^{R}=\int_{0}^{T}dt^{\prime }\frac{\Gamma_{L}\Gamma_{R}\left(n_{L}\left(t^{\prime }\right)-n_{R}\left(t^{\prime }\right)\right)}{K},$$

with $K\equiv \sum_{\nu =L,R}\Gamma_{\nu }\left(1+2n_{\nu }\left(t^{\prime }\right)\right)$, and

(26) $$G_{2}^{R}=\int \int dT_{L}dT_{R}\{\frac{2\Gamma_{L}\Gamma_{R}\left(\Gamma_{L}+\Gamma_{R}\right)}{K^{3}}\frac{dn_{R}}{dT_{R}}\frac{dn_{L}}{dT_{L}}\},$$

which coincide with the ones in Reference [28] and imply that the sum of $G_{1}^{\nu }$ and $G_{2}^{\nu }$ corresponds to the adiabatic contribution.

Considering this point, we find that the nonadiabatic contribution is described with a new extra term in $\langle \Delta q^{\nu }\rangle$ added to the adiabatic contribution in the form,

(27) $$\begin{array}{c} \langle \Delta q^{\nu }\rangle =G_{ad}^{\nu }+G_{nad}^{\nu }, \end{array}$$

with $G_{ad}^{\nu }=G_{1}^{\nu }+G_{2}^{\nu }$ and $G_{nad}^{\nu }=G_{3}^{\nu }$. This is consistent with the expression of $G_{3}^{\nu }$, which shows that, when $\rho_{00}\left(0\right)=\rho_{s,1}$ and the absolute value of $\Lambda_{i}\delta t$ is sufficiently large, we can neglect $G_{3}^{\nu }$. The former condition corresponds to the adiabatic approximation in Reference [28], where the population of the relevant system instantaneously approaches the steady state for the temperature setting at an initial time. The term $G_{3}^{\nu }$ shows that the nonadiabatic contribution to the transferred quantity explicitly depends on the initial condition of the relevant system, $\rho_{00}\left(0\right)$. Moreover, expanding Equation (13) about δt up to the first order, we find that the nonadiabatic effect described in $G_{3}^{\nu }$ shows a correction to both $G_{1}^{\nu }$ and $G_{2}^{\nu }$. In the following, we present a numerical evaluation of the formulas obtained.

### 3.4. Numerical Evaluation of the Nonadiabatic Spin Pumping

#### 3.4.1. Population Dynamics

**Figure 2:**
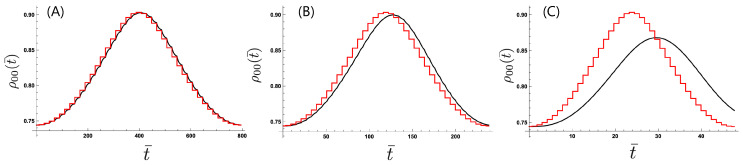
Time dependence of the population in the lower state of the two-level system with changing modulation frequency: (**A**) Ω=0.3THz, (**B**) Ω=1THz, and (**C**) Ω=5THz with s=0.01, $\omega_{c}=3\omega_{0}$, $\hbar \omega_{0}=25\;\mathrm{meV}$, and N=40. The time variable is scaled with $\omega_{0}$ as $\bar{t}=\omega_{0}t$.

Figure 2 presents the transient time evolution of $\rho_{00}\left(t\right)$ during the first period of modulation by changing the time interval δt while keeping the number of divisions *N* constant at N=40. We set parameters as λ=0.01, $\omega_{c}=3\omega_{0}$, and $\hbar \omega_{0}=25$ meV which shows the relaxation time ${\bar{\tau }}_{r}\equiv \omega_{0}\tau_{r}\approx 5$. (The value of $\hbar \omega_{0}$ is chosen to be the same as the typical value for a molecular junction in Reference [28].) Setting the initial condition of the two-level system with the effective inverse temperature as ${\bar{\beta }}_{s}=\frac{\hbar \omega_{0}}{k_{B}T_{s}}=\bar{\beta }\left(0\right)\left(\approx 1.07\right)$ corresponding to the stationary state for the initial temperature setting, we plot the time dependence of the population in the lower state, $\rho_{00}\left(t\right)$. The population $\rho_{00}\left(t\right)$ under the adiabatic approximation (Figure 2, red line) shows that the relevant system quickly approaches the stationary state corresponding to the temperature setting in each time interval. Setting the interval δt to be much larger than the relaxation time as in Figure 2A corresponding to the lower modulation frequency Ω=0.3 THz, we find that the relevant system mostly follows the temperature modulation as the stationary state is approached, which shows the feature close to the adiabatic approximation. With decreasing interval δt (Figure 2B,C), we find that the relevant system does not follow the temperature modulation thus exhibiting nonadiabaticity.

#### 3.4.2. Frequency Dependence

**Figure 3:**
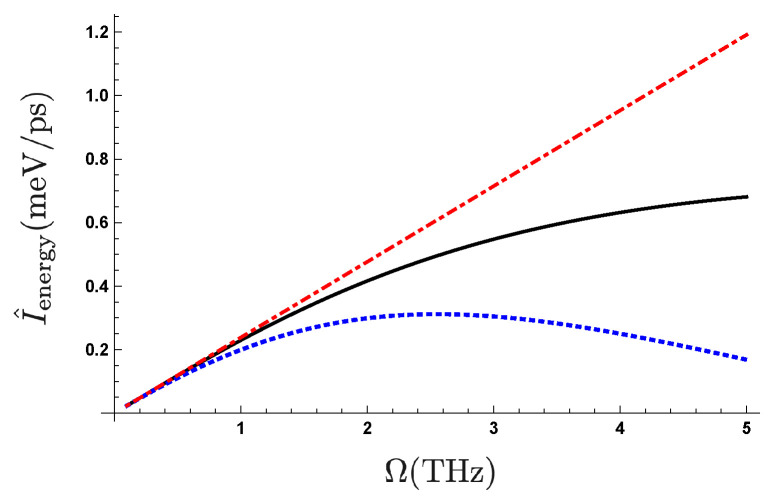
(Color online) Frequency dependence of pumped quantity $I_{\mathrm{energy}}$ with λ=0.01, $\omega_{c}=3\omega_{0}$, and $\hbar \omega_{0}=25\;\mathrm{meV}$ with changing initial conditions of the two-level system ${\bar{\beta }}_{s}$ values: (1) the black line corresponds to ${\bar{\beta }}_{s}=\bar{\beta }\left(0\right)\left(\approx 1.07\right)$ which is the effective inverse temperature of the stationary state for the initial temperature setting, (2) the blue dotted line to ${\bar{\beta }}_{s}=5$; (3) the red dottdashed line represents the frequency dependence of the net geometrical phase [28].

We show in Figure 3 the frequency dependence of the pumped quantity ${\hat{I}}_{\mathrm{energy}}=\frac{1}{T\hbar \omega_{0}}\left(\langle \Delta q^{R}\rangle -\langle \Delta q^{L}\rangle \right)$. For comparison, we also exhibit the frequency dependence of the quantity under the adiabatic approximation presented as a geometric phase in Reference [28]. We find that the nonadiabatic term decreases the pumped quantity in the higher frequency region. We also find that the pumped quantity depends on the initial condition of the two-level system. The feature shown in Figure 3 is universal for different settings of these parameters. For example, when we increase $\tau_{r}$ by decreasing the coupling strength with keeping the value of $\omega_{0}$, we find the similar feature of the frequency dependence ranging up to ∼10 GHz which corresponds to the maximum driving frequency of electronic voltage due to the limitation of experimental bandwidth at the present time. The parameter setting of λ in this study is chosen to expect the further acceleration of the recent rapid development of Tera Hz technology in a future.

## 4. Spin Pumping

### 4.1. A Minimum Model of Spin Pumping

**Figure 4:**
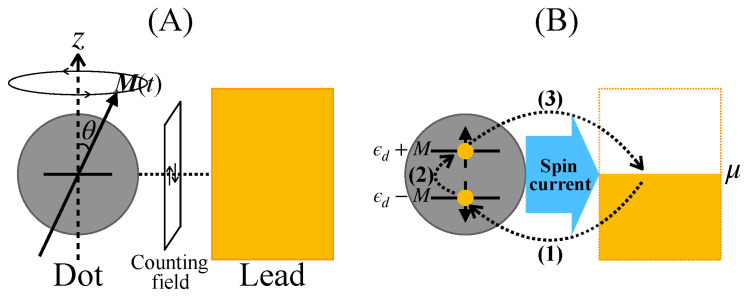
(**A**) The minimum model consists of a ferromagnetic quantum dot attached to an electron lead. The dot has a dynamic magnetization M(t) that rotates around the *z*-axis with a period T. The number of transferred electrons with spin magnetic moment ↑ (↓) is captured by the counting field. (**B**) Schematic of the spin current generation in the minimum model. The scheme can be summarized as follows: (1) an electron with ↓-spin enters from the lead onto the dot subject to the dot–lead interaction; (2) the spin of the electron is flipped by the precessing magnetization; (3) the electron with ↑-spin moves back from the dot to the lead.

We consider a minimum model of spin pumping involving a quantum dot with dynamic magnetization and an electron lead (Figure 4A). The magnetization of the dot M(t) rotates around the *z*-axis with a period T. An electron in the quantum dot is spin polarized because of the *s–d* exchange interaction with magnetization and is represented by the two-component creation and annihilation operators $d^{†}=\left(d_{↑}^{†},d_{↓}^{†}\right)$, and d, where ↑ and ↓ denote the direction of the electron’s spin magnetic moment parallel and antiparallel, respectively, to the *z*-axis.

The Hamiltonian of the minimum model consists of three terms $H\left(t\right)=H_{d}\left(t\right)+H_{l}+H_{t}$. $H_{d}\left(t\right)$, describing the dot, is defined by

(28) $$H_{d}\left(t\right)=d^{†}\left[\epsilon_{d}-M\left(t\right)\cdot \sigma \right]d,$$

where $\epsilon_{d}$ is the unpolarized energy of a dot electron, M(t)≡M(sinθsinϕ(t),sinθsinϕ(t),cosθ), and $\sigma =(\sigma_{x},\sigma_{y},\sigma_{z})$ the vector of Pauli matrices. Introducing the eigenstates $|j_{↑},j_{↓}\rangle$ (with $j_{↑(↓)}=0$ or 1) of the number operator of the dot electron $\sum_{\sigma }d_{\sigma }^{†}d_{\sigma }$ as a basis, the dot Hamiltonian is represented by the matrix

(29) $$\begin{array}{cc} & \begin{array}{cccc} |0,0\rangle & |0,1\rangle & |1,0\rangle & |1,1\rangle \end{array}\\ H_{d}\left(t\right)= & \left(\begin{array}{cccc} 0 & 0 & 0 & 0\\ 0 & \epsilon_{d}+M\cos\theta & -Me^{+i\phi (t)}\sin\theta & 0\\ 0 & -Me^{-i\phi (t)}\sin\theta & \epsilon_{d}-M\cos\theta & 0\\ 0 & 0 & 0 & 2\epsilon_{d} \end{array}\right) \end{array}.$$

The electron lead is described by the term

(30) $$H_{l}=\sum_{\sigma =↑,↓}\sum_{k}\epsilon_{k}c_{\sigma ,k}^{†}c_{\sigma ,k},$$

where $c_{\sigma ,k}$ and $c_{\sigma ,k}^{†}$ with σ=↑ or ↓ are annihilation and creation operators of a lead electron with energy $\epsilon_{k}$ and spin-σ. The dot–lead interaction is assumed to be spin conserving with

(31) $$H_{t}=\sum_{\sigma }\sum_{k}\hbar v_{k}\left(d_{\sigma }^{†}c_{\sigma ,k}+c_{\sigma ,k}^{†}d_{\sigma }\right),$$

where $\hbar v_{k}$ is the coupling strength, which we assume to be weak.

Intuitively, the generation of the spin current in the minimum model is summarized by the following scheme (see Figure 4B): (1) an electron with ↓-spin moves from lead to dot under the dot–lead interaction, (2) the spin of the electron is flipped by the precessing magnetization, and (3) an electron with ↑-spin moves back from dot to lead. For spin-current generation, the essential conditions required in setting parameter values are

(32) $$\epsilon_{d}-M<\mu <\epsilon_{d}+M\;\mathrm{and}\;\beta^{-1}\leq 2M,$$

where β is the inverse temperature of the lead.

### 4.2. FCS Formalism of the Spin Pumping

In the following, we apply the FCS outlined in Section 2 to evaluate the number of transferred electrons with spin σ from projective measurements of the electron number in the lead represented by $N_{\sigma }\equiv \sum_{k}c_{\sigma ,k}^{†}c_{\sigma ,k}$. By associating $H_{d}\left(t\right)$, $H_{l}$, and $H_{t}$ with $H_{S}$, $H_{E}$, and $H_{\mathrm{SE}}$, respectively, and defining an outcome of the projective measurement at time *t* as $n_{\sigma ,t}$, we analyze the electron dynamics under spin pumping.

In order to explicitly examine the influence of the relaxation process on the spin current generation, we discretize the rotation of M(t): divide the period T into *N* intervals, $t_{i}\leq t\leq t_{i+1}$(i=1,⋯,N) with $t_{1}=0$ and $t_{N+1}=T$; fix the direction of M(t) during each interval; and change ϕ at each $t_{i}$ discretely with substitution $\phi_{i}=\phi_{i-1}+\delta \phi$ with $\phi_{0}=0$, $\phi_{N}=2\pi$ and δϕ≡2π/N (see Note [57]). The net number of electrons with spin-σ during the *i*th interval can be evaluated from the difference in outcomes $\Delta n_{\sigma ,i}=n_{\sigma ,t_{i+1}}-n_{\sigma ,t_{i}}$.

By introducing counting fields $\lambda_{↑}$ and $\lambda_{↓}$ corresponding to observables $N_{↑}$ and $N_{↓}$, respectively, we can evaluate the mean value of transferred electrons,

(33) $$\langle \Delta n_{↑(↓),i}\rangle =\int_{t_{i}}^{t_{i+1}}J_{↑(↓)}\left(t\right),$$

with an inertial flow of electrons

(34) $$J_{↑(↓)}\left(t\right)\equiv \langle \;\langle 1|[\frac{\partial \Xi^{\left(\lambda_{↑(↓)}\right)}\left(t\right)}{\partial (i\lambda_{↑(↓)})}{]}_{\lambda_{↑(↓)}=0}|\rho^{\left(\lambda_{↑(↓)}=0\right)}\left(t\right)\rangle \;\rangle .$$

The inertial flow of electrons provides an instantaneous spin current,

(35) $$J_{\mathrm{spin}}\left(t\right)\equiv J_{↑}\left(t\right)-J_{↓}\left(t\right),$$

and its time integration over one period provides a temporal average of the spin current,

(36) $$I_{\mathrm{spin}}\equiv \frac{1}{T}\int_{0}^{T}J_{\mathrm{spin}}\left(t\right)dt.$$

To discuss the role of nonadiabaticity in spin pumping, we focus the Born-Markovian (long-time) limit by taking the limit t→∞ of the supermatrix $\Xi^{\left(\lambda \right)}\left(t\right)$ in each interval. In this limit, the matrix elements of $\Xi^{\left(\lambda \right)}$ are time-independent during each interval and determined by the direction of M in each interval.

### 4.3. Absence of Adiabatic Contribution

Let us first show absence of the adiabatic contribution to the spin pumping in the minimum model. In previous studies, the adiabatic regime of the spin pumping in the minimum model has been studied based on the linear expansion of the Green function in the rotation frequency of M(t) [23,24], which we referred to as the adiabaticity No. (2). The purpose of the present subsection is to re-examine the adiabatic contribution of the spin pumping from the view point of the adiabaticity No. (3), where we consider a sufficiently slow rotation of M(t) comparing to the relaxation time, that is, $\Omega \ll \tau_{r}^{-1}$ following the procedure by Sinitsyn and Nemenman in Reference [27].

Following the procedure, the adiabatic regime is assessed by dividing the cycle of modulation into time intervals δt(≡T/N) and assuming a quick approach of the system to its steady state in each interval. In the steady state of the minimum model, we can expect that the quantum dot is occupied by a single electron whose spin is aligned toward the direction of M(t), and, because of the rotational symmetry of the model, the steady state populations of the quantum dot are invariant under the rotation of M(t) around the *z*-axis. It indicates that no electron transfer occurs in the adiabatic regime. As a result, we can expect absence of the adiabatic contribution to the spin current generation. We provide an analytical proof of the intuitive observation in Appendix C (see also the original argument in Section 4 in Reference [58]).

As a result, we need to include the nonadiabatic effect to obtain a finite spin current. It is in marked contrast to the previous example of the energy pumping, where the adiabatic contribution to the energy pumping $G_{ad}^{\nu }$ is finite.

### 4.4. Numerical Evaluation of the Nonadiabatic Spin Pumping

We now turn to examine nonadiabaticity in spin pumping. For this purpose, we evaluate numerically the instantaneous spin current $J_{\mathrm{spin}}\left(t\right)$ and its temporal average $I_{\mathrm{spin}}$.

To describe the dot–lead coupling, we use the Ohmic spectral density with an exponential cutoff $v\left(\omega \right)\equiv \sum_{k}v_{k}^{2}\delta \left(\omega -\omega_{k}\right)=\lambda \omega \exp\left[-\omega /\omega_{c}\right]$, where λ is the coupling strength and $\omega_{c}$ is the cutoff frequency. For the numerical calculation, we chose 2M, the energy difference between the spin-↑ and -↓ states in the dot, as an energy unit. We distinguish parameters normalized by their units using an overbar (see Note [59]). Specific values of the normalized parameters are given in the figure captions. As we are focusing on the spin transfer driven by the rotating magnetization, the dot is set in a steady state Equation (A25) at $\bar{t}=0$ to exclude any transient spin transfer caused by the dot–lead contact.

#### 4.4.1. Electron Transfer Dynamics

**Figure 5:**
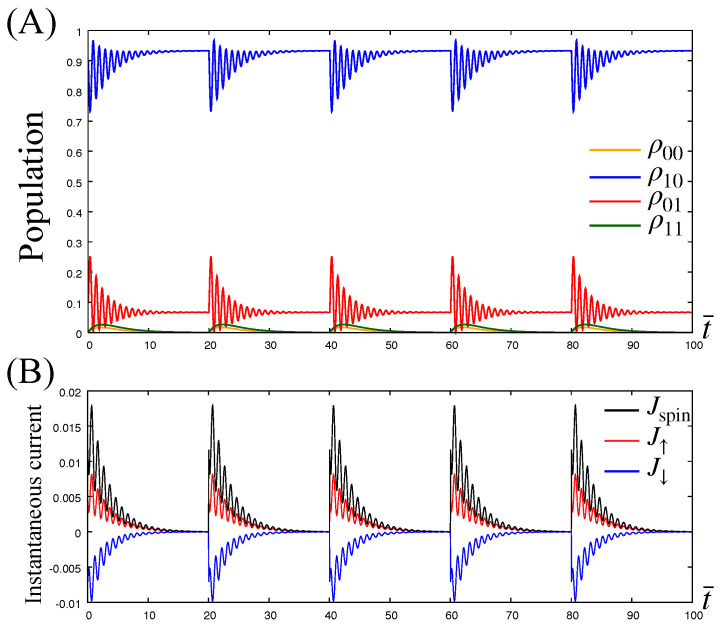
(**A**) Time evolution of the populations in the dot under the step-like precession of the magnetization with $\delta \bar{t}=20$ and N=5. The populations deviate from their steady-state values just after a sudden change of the angle ϕ, but then they approach new steady state-values for each $\phi_{i}$. The figure shows the steady-state values of the populations to be invariant. This is because of the rotational symmetry of the system about the *z*-axis. (**B**) The instantaneous electron and spin currents $J_{↑}\left(t\right)$ (red line), $J_{↓}\left(t\right)$ (blue line) and $J_{\mathrm{spin}}\left(t\right)$ (black line) corresponding to the population dynamics in panel (A). The time dependences of the instantaneous currents indicate that electrons starts moving between dot and lead just after the sudden change of ϕ, and $J_{↑}$ and $J_{↓}$ have opposing directions. The latter trend show that the instantaneous electron currents are balanced as a result of charge conservation in the lead. In contrast, the instantaneous spin current $J_{\mathrm{spin}}$ always takes positive values indicating constant spin current generation. The parameters are set to ${\bar{\epsilon }}_{d}=10$, $\bar{\mu }=10$, $\bar{\beta }=100$, λ=0.01, ${\bar{\omega }}_{c}=4$, θ=5π/6, and δϕ=2π/5, which satisfies the condition (32).

Let us first examine electron transfer dynamics under the cyclic rotation of the magnetization to show the generation of the spin current in the nonadiabatic regime. For this purpose, we numerically evaluate the time evolution of the populations $\rho_{jj^{\prime }}\left(t\right)=\langle j,j^{\prime }|\rho^{\left(0\right)}\left(t\right)|j,j^{\prime }\rangle$ ($\rho_{00}$: empty state, $\rho_{10}$: half-filled state with spin-↑, $\rho_{01}$: half-filled state with spin-↓, and $\rho_{11}$: completely filled state) and corresponding instantaneous electron and spin currents, $J_{↑(↓)}\left(t\right)$ and $J_{\mathrm{spin}}\left(t\right)$. In Figure 5A,B, we present the time evolution of populations and instantaneous currents for one cycle of the step-like rotation with division number N=5 and time interval δt=20. The change in angle at each subsequent $t_{i}$ is δϕ=2π/5, that is $\phi_{i}=\phi_{i-1}+2\pi /5$ with $\phi_{0}=0$.

In Figure 5A, we find that initially the populations deviate from their steady-state values by changing ϕ at $t_{i}$, but then they approach new steady-state values for each $\phi_{i}$ with the populations remaining unchanged from their initial values because the steady-state populations are independent of ϕ (see the analytic expression of the steady state, Equation (A25)). In the figure, the time evolution of the components $\rho_{01}$ and $\rho_{10}$ (blue and red lines) exhibit oscillations caused by transitions between states |0,1〉 and |1,0〉 in consequence of the applied magnetization M (Larmor precession). Its period is given by the inverse of the Larmor frequency $T_{L}\equiv h/2M=t_{u}$. The other two components $\rho_{00}$ and $\rho_{11}$ also exhibit transient behavior after changing ϕ but they do not exhibit a Larmor precession because the magnetization contributes transitions including neither |0,0〉 nor |1,1〉 (see Equation (29)).

In Figure 5B, the colored lines representing $J_{\sigma }$ show that spin-↑ electrons (red line) and spin-↓ electrons (blue line) are moving in opposite directions; the former move from dot to lead, whereas the latter move from lead to dot. These trends show that the instantaneous electron currents $J_{↑}$ and $J_{↓}$ are balanced as a result of charge conservation in the lead. In contrast, the instantaneous spin current (black line) always takes positive values, $J_{\mathrm{spin}}>0$, indicating the generation of positive spin current into the lead without an associated charge current, which we call pure spin current.

#### 4.4.2. Frequency Dependence

We next consider the dependence of the spin current on the frequency of precession Ω=2π/T. Here we change the period of precession T=Nδt by varying the time interval δt while the number of divisions remains fixed to N=20. All other parameters and initial conditions are set as before.

**Figure 6:**
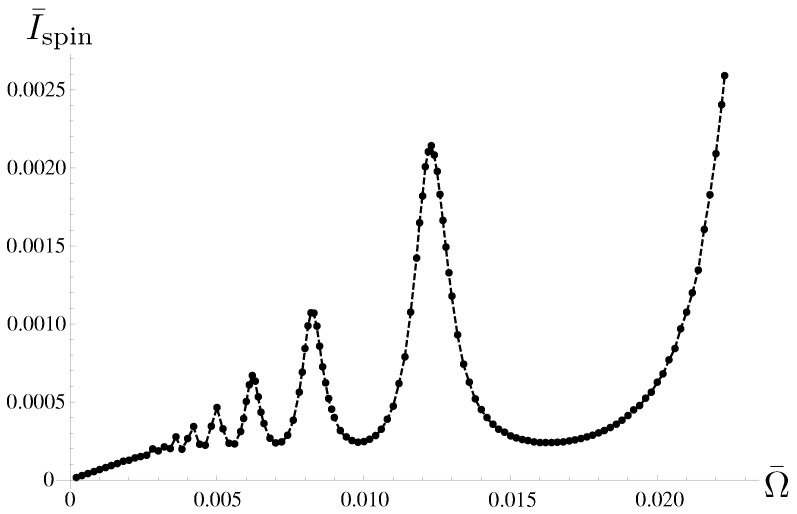
Frequency dependence of the temporal average of spin current $I_{\mathrm{spin}}$. The division number of the step-like precession is now set to N=20. With fixed δϕ=π/10, the frequency is changed by changing δt. The frequency dependence exhibits two characteristic features: the spin current depends linearly on $\bar{\Omega }$ for $\bar{\Omega }≲0.0025$, whereas it exhibits oscillation with respect to $\bar{\Omega }$ for $\bar{\Omega }≳0.0025$. The other parameters are the same as in Figure 5.

In Figure 6, we plot the dependence of the averaged spin current $I_{\mathrm{spin}}$ against the normalized frequency $\bar{\Omega }\equiv \Omega /\omega_{u}$.

The frequency dependence of $I_{\mathrm{spin}}$ features two characteristic regimes: a low-frequency regime, where $I_{\mathrm{spin}}$ depends linearly on Ω ($\bar{\Omega }≲0.0025$) and a high-frequency regime, where $I_{\mathrm{spin}}$ exhibits oscillations with respect to Ω. These characteristics are explained by comparing the time interval $\delta \bar{t}$ and the relaxation time ${\bar{\tau }}_{r}$ of the population of dot electrons (${\bar{\tau }}_{r}\approx 5$ in the present case; see Figure 5A). For lower frequencies, for which $\delta \bar{t}\gg {\bar{\tau }}_{r}$, the numerator of the time integral of $J_{\mathrm{spin}}\left(t\right)$ in Equation (36) becomes constant because the instantaneous spin current has already vanished at a certain $\bar{t}≲\delta \bar{t}$ (see Figure 5A), which results in the linear dependence of $I_{\mathrm{spin}}$ on $\bar{\Omega }$. As $\bar{\Omega }$ becomes larger and the time interval satisfies $\delta \bar{t}≲{\bar{\tau }}_{r}$, the angle ϕ changes during the relaxation process. In this situation, the electron dynamics exhibits two extreme features; when $\delta \bar{t}$ is an integer multiple of the period of the Larmor precession h/2M, we have resonance enhancement of the transition between half-filled states |0,1〉 and |1,0〉 by the sudden change of ϕ to exhibit a maximum of $I_{\mathrm{spin}}$, whereas it is anti-resonantly suppressed to exhibit a minimum when $\delta \bar{t}$ is a half-integer multiple of the period [58].

Calculating the spin current for different values of θ, we find that the spin polarization of the spin current exhibits a dependence on θ in that for 0<θ<π/2 the spin polarization is antiparallel to the *z*-axis, whereas for π/2<θ<π the spin polarization is parallel to the *z*-axis. For θ=0,π/2,π, the spin current vanishes because the spin flip in the quantum dot does not occur for θ=0,π or the two half-filled states in the dot |1,0〉 and |0,1〉 degenerate for θ=π/4 (see Equation (29)).

Finally, we note that the averaged spin current $I_{\mathrm{spin}}$ diverges with respect to $\bar{\Omega }$. The divergence is caused by the accumulation of a nonzero impetus of current $J_{\mathrm{spin}}\left(t\right)$ just after the sudden change of ϕ (see Figure 5). In Reference [60], we showed that the nonzero impetus of $J_{\mathrm{spin}}\left(t\right)$ is an unphysical effect caused by the Born-Markovian approximation, and the divergence is eliminated by taking into account the non-Markovian effect by keeping the upper bound of the time integration in (10) finite.

## 5. Discussion and Conclusions

In this paper, we reviewed studies which go beyond the conventional adiabatic approximation for open quantum systems to transfer energy quanta and electron spins with using the full counting statistics, which could provide conditions to show quicker transport. We considered a setup consisting of a two-level system representing an anharmonic junction or a quantum dot and its environment(s) representing a canonical or grand canonical ensemble of the energy quanta and the electron to be transferred. We needed to take into account relaxation phenomena in discussing the transfer. In this case, the adiabatic approximation corresponded to the situation where the relevant system such as the two-level system approaches its stationary state faster than the period of modulation, that is, $\tau_{r}^{-1}\gg \Omega$ with $\tau_{r}$ the relaxation time of the two-level system and Ω the modulation frequency. Because the relaxation time is finite, the condition for which the adiabatic approximation is valid corresponds to the much longer period of the modulation than $\tau_{r}$. This means that we can analyze systematic features including adiabatic as well as nonadiabatic features by changing the ratio of the modulation period and $\tau_{r}$. To clarify the relationship between modulation period and $\tau_{r}$, we discretized the external modulation thereby permitting a systematic analysis of the ratio by changing each interval while retaining the validity of the Born–Markov approximation. For energy quanta pumping, we showed that the nonadiabatic effect contributes a new term to the formula for the pumped quantity under the adiabatic approximation. For spin pumping, we showed that adiabaticity made no contribution but nonadiabaticity is essential. Comparing these features, we showed that the adiabatic contribution can vanish when the stationary state does not depend on the external modulation as for spin pumping. This means that we need to pay attention to the feature of the stationary state in using the adiabatic approximation in describing relaxation phenomena. (We would draw the reader’s attention to the differences in the meaning of nonadiabaticity which has been used in the electron charge pumping by modulation of single gate voltage [61,62].)

With the same setup, the role of nonadiabaticity in pumping phenomena involving energy quanta was discussed more extensively under continuous modulation [63] where the relaxation of the two-level system is treated within the Born–Markovian approximation. In recent work of the present authors on the role of the non-Markovian effect on spin pumping phenomena [60], we found that a nonzero impetus of the dynamics of the pumped quantity under the Born–Markovian approximation shows an unphysical effect, especially for higher modulation frequencies or for the short time regimes. Because the instantaneous impetus contributes strongly under continuous modulation, including the non-Markovian effect would also be necessary in pumping phenomena of energy quanta, especially in evaluating the feature under continuous modulation. This situation remains an open problem. In addition, we described in this work the relaxation process with ordered cumulants of up to second order in the system–environment interaction. An extension to higher orders of cumulants is necessary if we are to discuss relaxation phenomena under strong system–environment interactions. The inclusion of cumulants up to infinite order within the Markovian approximation has been discussed for spin pumping phenomena within the linear response regime using the Green functions [19]. To discuss the non-Markovian effect, it would be necessary to include higher-order cumulants, a topic that remains for a future study.

We can find recent extensions of the treatments with full counting statistics into the strong system-environment coupling for heat transfer [64] and electron pumping [65]. The essential idea to go beyond the weak coupling is to use the similarity (unitary) transformations: the polaron transformation (the reaction coordinate mapping) is used in the former (latter) studies, respectively. As mentioned in the general formalism of FCS, it should be noted that we need careful treatments on the joint probability, Equation (2), when we use the similarity (unitary) transformations on the time evolution operator. The transformation of the projective measurement is also necessary to recover the original joint probability (See Reference [45]). It might be necessary to compare the dynamics of transported quantity with and without the transformation of the projective measurement.

Since the treatment of FCS to discuss the nonadiabatic effects on quantum pumping is general, we can apply it to many other cases: One of the most interesting issues is to study the non-adiabatic treatment on the combined effect caused by multiple external parameters such as in References [66,67,68] where adiabatic transport of charge and/or heat is discussed under time-dependent potential and two reservoirs with biased potentials. We can find other issues to remove the adiabatic approximation in spin pumping via a quantum dot between reservoirs with biased chemical potentials [69] and in the quantum transport and/or quantum pumping under dynamical motion of quantum dot [70] based on the recent developments of experimental techniques on microelectromechanical systems [71]. Further, it would be interesting to discuss the non-adiabatic effect on ac-driven electron systems coupled to multiple reservoirs at finite temperature whose adiabatic treatment is discussed in Reference [72]. We expect that these treatments could provide insights to find new applications, such as the design of nanomachines and understanding of the quantum thermodynamics, as well as quicker transport.

## Appendix A. Derivation of Quantum Master Equation for FCS

When we consider the FCS [44], the density operator $W^{\left(\lambda \right)}\left(t\right)$ evolves in time in accordance with the modified Liouville–von Neumann equation

(A1) $${\dot{W}}^{\left(\lambda \right)}\left(t\right)=-iL^{\left(\lambda \right)}W^{\left(\lambda \right)}\left(t\right),$$

where $L^{\left(\lambda \right)}$ is the Liouville operator defined as $L^{\left(\lambda \right)}A=\frac{1}{\hbar }\left[H_{\lambda }A-AH_{-\lambda }\right]\equiv \frac{1}{\hbar }{\left[H,A\right]}_{\lambda }$ for arbitrary operator *A* with $H_{\lambda }=e^{\left(i/2\right)\lambda H_{E}}He^{-\left(i/2\right)\lambda H_{E}}=H_{0}+H_{SE,\lambda }$. With these relations, L is divided into

(A2) $$L^{\left(\lambda \right)}=L_{0}^{\left(\lambda \right)}+L^{\prime (\lambda )}$$

where

(A3) $$L_{0}^{\left(\lambda \right)}A=\frac{1}{\hbar }\left[H_{0}A-AH_{0}\right]\;\;,\;\;L^{\prime (\lambda )}A=\frac{1}{\hbar }\left[H_{SE,\lambda }A-AH_{SE,-\lambda }\right].$$

We eliminate the variables of the environment using a projection operator P, which satisfies the idempotent relation, $P^{2}=P$. We also introduce a complementary operator Q≡1−P. Denoting the relevant and irrelevant parts of the time evolution operator as [52]

(A4) $$x\left(t\right)\equiv Pe^{-iL^{\left(\lambda \right)}t},\;\;\;y\left(t\right)\equiv Qe^{-iL^{\left(\lambda \right)}t},$$

with an initial time $t_{0}=0$, we obtain

(A5) $$\frac{d}{dt}x\left(t\right)=P\left(-iL^{\left(\lambda \right)}\right)x\left(t\right)+P\left(-iL^{\left(\lambda \right)}\right)y\left(t\right)\;,$$

and

(A6) $$\frac{d}{dt}y\left(t\right)=Q\left(-iL^{\left(\lambda \right)}\right)x\left(t\right)+Q\left(-iL^{\left(\lambda \right)}\right)y\left(t\right)\;.$$

The formal solution of Equation (A6) is given by

(A7) $$\begin{array}{ccc} y(t) & = & \int_{0}^{t}e^{-QiL^{\left(\lambda \right)}\left(t-\tau \right)}Q\left(-iL^{\left(\lambda \right)}\right)x\left(\tau \right)d\tau +e^{-QiL^{\left(\lambda \right)}t}Q. \end{array}$$

Using $x\left(\tau \right)=Pe^{iL^{\left(\lambda \right)}\left(t-\tau \right)}e^{-iL^{\left(\lambda \right)}t}=Pe^{iL^{\left(\lambda \right)}\left(t-\tau \right)}\left(x\left(t\right)+y\left(t\right)\right)$ and

(A8) $$\theta \left(t\right)=1-\int_{0}^{t}e^{-QiL^{\left(\lambda \right)}\tau }Q\left(-iL^{\left(\lambda \right)}\right)Pe^{iL^{\left(\lambda \right)}\tau }d\tau \equiv 1-\sigma \left(t\right),$$

we rewrite the formal solution of y(t) in the form

(A9) $$y\left(t\right)=\theta {\left(t\right)}^{-1}\left(\left(1-\theta \left(t\right)\right)x\left(t\right)+e^{-QiL^{\left(\lambda \right)}t}Q\right).$$

By substituting Equation (A9) into Equation (A5), we obtain

(A10) $$\frac{d}{dt}x\left(t\right)=P\left(-iL^{\left(\lambda \right)}\right)\theta {\left(t\right)}^{-1}x\left(t\right)+P\left(-iL^{\left(\lambda \right)}\right)\theta {\left(t\right)}^{-1}e^{Q(-iL^{\left(\lambda \right)})t}Q,$$

which holds for arbitrary projection operator and initial condition. Using the relation $\theta {\left(t\right)}^{-1}=\sum_{n=0}^{\infty }\sigma {\left(t\right)}^{n}$, the first term on the right-hand side of Equation (A10) is rewritten as

(A11) $$P\left(-iL^{\left(\lambda \right)}\right)\theta {\left(t\right)}^{-1}x\left(t\right)=P\left(-iL^{\left(\lambda \right)}\right)x\left(t\right)+P\left(-iL^{\left(\lambda \right)}\right)\sigma \left(t\right)x\left(t\right)+\cdots .$$

To pick out the lower order of $L^{\prime (\lambda )}$, we use the relation

(A12) $$e^{-QiL^{\left(\lambda \right)}t}Q=e^{-iL_{0}^{\left(\lambda \right)}t}QT_{+}\exp[\int_{0}^{t}dt^{\prime }e^{iL_{0}^{\left(\lambda \right)}t^{\prime }}Q\left(-iL^{\prime (\lambda )}\right)Qe^{-iL_{0}^{\left(\lambda \right)}t^{\prime }}],$$

and $PL_{0}^{\left(\lambda \right)}=L_{0}^{\left(\lambda \right)}P$, which gives

(A13) $$\begin{array}{ccc} P\left(-iL^{\left(\lambda \right)}\right)\theta {\left(t\right)}^{-1}x\left(t\right) & = & P\left(-iL^{\left(\lambda \right)}\right)x\left(t\right)+P\left(-iL^{\prime (\lambda )}\right)\int_{0}^{t}e^{-iL_{0}^{\left(\lambda \right)}\tau }Q\left(-iL^{\prime (\lambda )}\right)Pe^{iL_{0}^{\left(\lambda \right)}\tau }d\tau x\left(t\right)+\cdots \\ \; & = & P\left(-iL^{\left(\lambda \right)}\right)x\left(t\right)+P\left(-iL^{\prime (\lambda )}\right)\int_{0}^{t}Q\left(-iL^{\prime (\lambda )}\left(-\tau \right)\right)Pd\tau x\left(t\right)+\cdots , \end{array}$$

where we have used the definition

(A14) $${\hat{L}}_{1}^{\left(\lambda \right)}\left(t\right)=e^{iL_{0}^{\left(\lambda \right)}t}L^{\prime (\lambda )}e^{-iL_{0}^{\left(\lambda \right)}t}.$$

When we multiply the initial condition $W^{\left(\lambda \right)}\left(0\right)$ by the right-hand side of Equation (A8), we obtain the TCL equation for reduced density operator under FCS.

Let us consider a projection operator $P=\rho_{E}{\mathrm{Tr}}_{E}$ where ${\mathrm{Tr}}_{E}$ refers to a trace operation over the environment. When we multiply the initial condition of the density operator of the total system, W(λ,0) from the right by $x_{S}\left(t\right)$ in Equation (A2), we obtain

(A15) $$x\left(t\right)W^{\left(\lambda \right)}\left(0\right)=\rho_{E}{\mathrm{Tr}}_{E}W^{\left(\lambda \right)}\left(t\right)$$

Defining ${\mathrm{Tr}}_{E}W^{\left(\lambda \right)}\left(t\right)\equiv \rho^{\left(\lambda \right)}\left(t\right)$, we obtain the TCL equation for the reduced density operator under FCS,

(A16) $$\frac{d}{dt}\rho^{\left(\lambda \right)}\left(t\right)={\mathrm{Tr}}_{E}\left[\left(-iL\right)\rho^{\left(\lambda \right)}\left(t\right)\right]+\zeta^{\left(\lambda \right)}\left(t\right)\rho^{\left(\lambda \right)}\left(t\right)+\psi^{\left(\lambda \right)}\left(t\right),$$

with

(A17) $$\begin{array}{ccc} \zeta^{\left(\lambda \right)}\left(t\right) & \equiv & \sum_{n=2}^{\infty }\xi_{n}^{\left(\lambda \right)}\left(t\right), \end{array}$$

(A18) $$\begin{array}{ccc} \psi^{\left(\lambda \right)}\left(t\right) & \equiv & {\mathrm{Tr}}_{E}\left[\left(-iL\right)\theta {\left(t\right)}^{-1}e^{Q(-iL)t}QW^{\left(\lambda \right)}\left(0\right)\right]. \end{array}$$

Equation (9) with (10) is obtained by taking the lower term of $\xi^{\left(\lambda \right)}\left(t\right)$ up to second order in $L^{\prime }$ and replacing $\xi_{2}^{\left(\lambda \right)}\left(t\right)$ with $\xi^{\left(\lambda \right)}\left(t\right)$,

(A19) $$\xi^{\left(\lambda \right)}\left(t\right)\rho \left(\lambda ,t\right)=\int_{0}^{t}{\mathrm{Tr}}_{E}\left[\left(-iL^{\prime }\right)Q\left(-iL^{\prime }\left(-\tau \right)\right)\rho_{E}\rho \left(\lambda ,t\right)\right]d\tau .$$

with the factorized initial condition of the total system as $W^{\left(\lambda \right)}\left(0\right)=\rho_{E}\rho^{\lambda }\left(0\right)$, which makes the third term on the right-hand side of Equation (A16) vanish. With the assumption ${\mathrm{Tr}}_{E}H_{SE,\lambda }=0$, we have

(A20) $$\begin{array}{ccc} \xi^{\left(\lambda \right)}\left(t\right)\rho^{\left(\lambda \right)}\left(t\right)\\ \; & \; & \;={\left(\frac{i}{\hbar }\right)}^{2}{\mathrm{Tr}}_{E}{\left[H_{SE}{\left[H_{SE}\left(-\tau \right),\rho_{E}\rho^{\left(\lambda \right)}\left(t\right)\right]}_{\lambda }\right]}_{\lambda }],\\ \; & \; & \;={\left(\frac{i}{\hbar }\right)}^{2}{\mathrm{Tr}}_{E}[H_{SE,\lambda }H_{SE,\lambda }\left(-\tau \right)\rho_{E}\rho^{\left(\lambda \right)}\left(t\right)-H_{SE,\lambda }\rho_{E}\rho^{\left(\lambda \right)}\left(t\right)H_{SE,-\lambda }\left(-\tau \right)\\ \; & \; & \;-H_{SE,\lambda }\left(-\tau \right)\rho_{E}\rho^{\left(\lambda \right)}\left(t\right)H_{SE,-\lambda }+\rho_{E}\rho^{\left(\lambda \right)}\left(t\right)H_{SE,-\lambda }\left(-\tau \right)H_{SE,-\lambda }], \end{array}$$

which coincides with Equation (110) in [44].

## Appendix B. Connection between Two Formalisms of the FCS

We next explain the relationship between the two formalisms of the FCS provided by Esposito et al. in Reference [44] and by Sinitsyn and Nemenman in Reference [27]. To establish the connection between the two formalisms, we consider the number of quanta *N* as the observable to be measured to identify the number of quantum transfers from S to E, as stipulated in Reference [27]. For a large class of open quantum systems, the system–environment interaction is described by an interaction Hamiltonian of the form $H_{\mathrm{SE}}=V_{+}+V_{-}\equiv A⊗B^{†}+A^{†}⊗B$, where $B^{†}$ and *B* are creation and annihilation operators of a quanta in the environment, respectively, and *A* is either a Hermitian or non-Hermitian operator acting on the relevant system. $V_{+}\equiv A⊗B^{†}$ or $V_{-}=V_{+}^{†}$ describes transfer of a quanta from S to E or from E to S. In this instance, the number operator of the quanta is given by $N=B^{†}B$. The generalized Liouvillian is expressed as $L^{\left(\lambda \right)}=L_{0}+L_{+}e^{i\lambda /2}+L_{-}e^{-i\lambda /2}$, where $L_{0}W^{\left(\lambda \right)}\equiv \hbar^{-1}\left(H_{0}W^{\left(\lambda \right)}-W^{\left(\lambda \right)}H_{0}\right)$ is the unperturbed Liouvillian, $L_{+}W^{\left(\lambda \right)}\equiv \hbar^{-1}\left(V_{+}W^{\left(\lambda \right)}-W^{\left(\lambda \right)}V_{+}^{†}\right)$ and $L_{-}W^{\left(\lambda \right)}\equiv \hbar^{-1}\left(V_{-}W^{\left(\lambda \right)}-W^{\left(\lambda \right)}V_{-}^{†}\right)$ are Liouvillians describing the transfer of a quanta from S to E and from E to S, respectively. Using the formal solution of Equation (8) with the given Liouvillian in Equation (6), we obtain a formal expression for the moment generating function

(A21) $$Z\left(\lambda \right)=\sum_{n=-\infty }^{\infty }P_{n}e^{in\lambda },$$

with $P_{n}$ the probability of having *n* net transitions from S to E, e.g.,

(A22) $$\begin{array}{c} \begin{array}{ll} P_{1}=\mathrm{Tr}[( & \int_{t_{i}}^{t}dt_{1}U_{0}\left(t,t_{1}\right)\left(-iL_{+}\right)U_{0}\left(t_{1},t_{i}\right)+\int_{t_{i}}^{t}dt_{3}\int_{t_{i}}^{t_{3}}dt_{2}\int_{t_{i}}^{t_{2}}dt_{1}U_{0}\left(t,t_{3}\right)\left(-iL_{+}\right)\\ & \times U_{0}\left(t_{3},t_{2}\right)\left(-iL_{-}\right)U_{0}\left(t_{2},t_{1}\right)\left(-iL_{+}\right)U_{0}\left(t_{1},t_{i}\right)\cdots )\bar{W}\left(t_{i}\right)], \end{array} \end{array}$$

where $U_{0}\left(t_{1},t_{2}\right)=\exp\left[-iL_{0}\left(t_{1}-t_{2}\right)\right]$ is the unperturbed time evolution operator. The expression for the moment generating function corresponds to Equation (9) in Reference [27].

## Appendix C. An Analytical Proof of the Absence of Adiabatic Contribution to the Spin Pumping

Following the procedure by Sinitsyn and Nemenman in Reference [27], we divide the cycle of precession into intervals δt, which correspond to the step-like changes of M. For the step-like precession, the density matrix of the dot during $t_{i}\leq t\leq t_{i+1}$ is given by

(A23) $$|\rho_{i}^{\left(0\right)}\left(t\right)\rangle \;\rangle =e^{\Xi_{i}^{\left(0\right)}\left(t-t_{i}\right)}\prod_{j=1}^{i-1}e^{\Xi_{j}^{\left(0\right)}\delta t}|\rho_{1}^{\left(0\right)}\left(0\right)\rangle \;\rangle ,$$

where we denote the density matrix and the generator in the *i*th interval as $|\rho_{i}^{\left(0\right)}\left(t\right)\rangle \;\rangle$ and $\Xi_{i}^{\left(0\right)}$, respectively, and $|\rho_{1}^{\left(0\right)}\left(0\right)\rangle \;\rangle$ is the initial condition in the first interval. Taking the spectral decomposition of $\Xi_{i}^{\left(0\right)}$ and assuming a quick approach of the system to its steady state in each interval in Equation (A23), as in Reference [27], the terms remaining in the decomposition are those that contain the steady state $|u_{0}^{0}\left(t_{i}\right)\rangle \;\rangle$ satisfying $\Xi_{i}^{\left(0\right)}|u_{0}^{0}\left(t_{i}\right)\rangle \;\rangle =0$. Evaluating the density matrix up to first order in δt, we find the first-order term vanishes, implying the invariance of the steady state populations of the dot under the step-like rotation of ϕ in our model (see Appendix D in Reference [58]). Thus, we find that the density matrix at time *t* under the adiabatic limit can be approximated by the steady state as $|\rho_{0}^{\left(0\right)}\left(t\right)\rangle \;\rangle \approx |u_{0}^{\left(0\right)}\left(t_{i}\right)\rangle \;\rangle$. For this steady state $|u_{0}^{\left(0\right)}\left(t_{i}\right)\rangle \;\rangle$ given by Equation (A25), we also find that there is no electron transfer between dot and lead; specifically, we find that

(A24) $$\langle \Delta n_{\sigma ,i}\rangle \approx \int_{t_{i}}^{t_{i+1}}dt^{\prime }\langle \;\langle 1|[\frac{\partial \Xi_{i}^{\left(\lambda_{\sigma }\right)}}{\partial (i\lambda_{\sigma })}{]}_{\lambda_{\sigma }=0}|u_{0}^{\left(0\right)}\left(t_{i}\right)\rangle \;\rangle =0,$$

indicating that there is no net electron transfer in the interval and hence the generated spin current represented by Equation (36) is totally absent in the adiabatic limit. We therefore need to include the nonadiabatic effect to generate a finite spin current. The result is in marked contrast to the previous example of the energy pumping, where the adiabatic contribution to the energy pumping $G_{ad}^{\nu }$ is finite.

## Appendix D. Steady State of the Minimum Model

The steady state $|u_{0}^{\left(\lambda_{\sigma }=0\right)}\left(t_{i}\right)\rangle \;\rangle$, satisfying $\Xi_{i}^{\left(\lambda_{\sigma }=0\right)}|u_{0}^{\left(\lambda_{\sigma }=0\right)}\left(t_{i}\right)\rangle \;\rangle =0$ is analytically obtained using a graphical method discussed in Reference [73]. In Appendix C of Reference [58], we provide a detailed derivation of the steady state. For use in the present paper, here we simply present the result.

The dynamics of the populations described by the TCL master equation is closed for the six components of the reduced density matrix, $\rho_{00}\left(t\right)\equiv \langle 0,0|\rho^{\left(\lambda_{\sigma }=0\right)}\left(t\right)|0,0\rangle$, $\rho_{01}\left(t\right)\equiv \langle 0,1|\rho^{\left(\lambda_{\sigma }=0\right)}\left(t\right)|0,1\rangle$, $\rho_{0110}\left(t\right)\equiv \langle 0,1|\rho^{\left(\lambda_{\sigma }=0\right)}\left(t\right)|1,0\rangle$, $\rho_{1001}\left(t\right)\equiv \langle 1,0|\rho^{\left(\lambda_{\sigma }=0\right)}\left(t\right)|0,1\rangle$, $\rho_{10}\left(t\right)\equiv \langle 1,0|\rho^{\left(\lambda_{\sigma }=0\right)}\left(t\right)|1,0\rangle$, and $\rho_{11}\left(t\right)\equiv \langle 1,1|\rho^{\left(\lambda_{\sigma }=0\right)}\left(t\right)|1,1\rangle$. By arranging these connected components as $|\rho^{\left(\lambda_{\sigma }=0\right)}\left(t\right)\rangle \;\rangle ={\left[\rho_{00}\left(t\right),\rho_{01}\left(t\right),\rho_{0110}\left(t\right),\rho_{1001}\left(t\right),\rho_{10}\left(t\right),\rho_{11}\left(t\right)\right]}^{t}$, where ${\left[\cdots \right]}^{t}$ denotes transposition, an analytic expression of its steady state obtains,

(A25) $$|u_{0}^{\left(\lambda_{\sigma }=0\right)}\left(t_{i}\right)\rangle \;\rangle =\left(\begin{array}{c} f^{-}\left(\epsilon_{↑}\right)f^{-}\left(\epsilon_{↓}\right)\\ \cos^{2}\frac{\theta }{2}f^{+}\left(\epsilon_{↑}\right)f^{-}\left(\epsilon_{↓}\right)+\sin^{2}\frac{\theta }{2}f^{-}\left(\epsilon_{↑}\right)f^{+}\left(\epsilon_{↓}\right)\\ e^{+i\phi_{i}}\cos\frac{\theta }{2}\sin\frac{\theta }{2}\left[f^{+}\left(\epsilon_{↑}\right)f^{-}\left(\epsilon_{↓}\right)-f^{-}\left(\epsilon_{↑}\right)f^{+}\left(\epsilon_{↓}\right)\right]\\ e^{-i\phi_{i}}\cos\frac{\theta }{2}\sin\frac{\theta }{2}\left[f^{+}\left(\epsilon_{↑}\right)f^{-}\left(\epsilon_{↓}\right)-f^{-}\left(\epsilon_{↑}\right)f^{+}\left(\epsilon_{↓}\right)\right]\\ \sin^{2}\frac{\theta }{2}f^{+}\left(\epsilon_{↑}\right)f^{-}\left(\epsilon_{↓}\right)+\cos^{2}\frac{\theta }{2}f^{-}\left(\epsilon_{↑}\right)f^{+}\left(\epsilon_{↓}\right)\\ f^{+}\left(\epsilon_{↑}\right)f^{+}\left(\epsilon_{↓}\right) \end{array}\right),$$

where $f^{+}\left(\epsilon_{k}\right)\equiv {\mathrm{Tr}}_{l}\left[c_{\sigma ,k}^{†}c_{\sigma ,k}\rho_{l}^{\mathrm{eq}}\right]$, $f^{-}\left(\epsilon_{k}\right)\equiv {\mathrm{Tr}}_{l}\left[c_{\sigma ,k}c_{\sigma ,k}^{†}\rho_{l}^{\mathrm{eq}}\right]$, $\epsilon_{↑}\equiv \epsilon_{d}-M$ and $\epsilon_{↓}\equiv \epsilon_{d}+M$. From the expression, we find that the steady-state values of the populations $\rho_{00}$, $\rho_{01}$, $\rho_{10}$ and $\rho_{11}$ are independent of angle ϕ. Thus, the steady state populations remain unchanged by changing ϕ.
